# A novel optineurin genetic mutation associated with open-angle glaucoma in a Chinese family

**Published:** 2009-08-20

**Authors:** Zheng Xiao, Qingfeng Meng, James C. Tsai, Huiping Yuan, Na Xu, Yuanyuan Li

**Affiliations:** 1 Department of Ophthalmology, Second Affiliated Hospital of Harbin Medical University, Heilongjiang, Harbin, China; 2 Department of Ophthalmology and Visual Science, School of Medicine, Yale University, New Haven, CT

## Abstract

**Purpose:**

To identify the genetic mutation associated with distinct cases of open-angle glaucoma noted in a Chinese family.

**Methods:**

Clinical examination and pedigree analysis were undertaken in a family with a large number of primary open-angle glaucoma cases. Venous blood samples were drawn from six affected and six unaffected subjects in the family. Genomic DNA was extracted. Linkage to the optineurin gene (*OPTN*) locus was not excluded. Potential mutation in *OPTN* was screened by polymerase chain reaction (PCR) analysis of its exon regions and direct sequencing.

**Results:**

A missense mutation, A1274G, in exon 10 of *OPTN* was identified in affected patients of the family. The corresponding amino acid change was Lys322Glu. This mutation was not found in unaffected family members of the family or in 87 unrelated normal controls.

**Conclusions:**

A novel mutation of a Lys322Glu change in *OPTN* is responsible for this familial case of primary open-angle glaucoma observed in northeastern China.

## Introduction

Over the past century, there has been increasing awareness of a genetic predisposition in the development of primary open-angle glaucoma (POAG) [1]. In 1997, the myocilin gene (*MYOC*), formerly referred to as the trabecular meshwork-induced glucocorticoid response protein (*TIGR*), was mapped to 1q, the locus associated with juvenile open-angle glaucoma [2]. In 2002, mutations in the optineurin gene (*OPTN*) were found in a large British pedigree with autosomal dominant normal tension glaucoma (NTG). At the present time, more than 20 distinct mutations have been found in the *OPTN* coding region, four of which, having close correlation with POAG, are located in exons 4, 5, 6, and 16 [3]. *OPTN* mutations have been identified only in Hong Kong and Guangzhou, China [4]. In this study, we sought to analyze a cohort of patients in northeastern China with familial POAG for mutations in *OPTN*.

## Methods

### Clinical evaluation

The respective patients were evaluated in the Department of Ophthalmology at the Second Affiliated Hospital of Harbin Medicine University(Harbin, China). Informed consent was obtained from all participants in accordance with the Declaration of Helsinki, and the study was approved by the Heilongjiang Institutional Review Board.

A diagnosis of POAG was made in patients if they had the following criteria: 1) at least one of their intraocular pressure (IOP) readings was more than 21 mmHg; 2) the patients must have open anterior chamber and were exclusive of secondary factors (e.g. pigmentary findings); and 3) the presence of typical glaucomatous cupping of the optic disc with visual field defects.

### Genotyping and linkage analysis

Two microsatellite markers closely linked to two known loci were used to perform allele-sharing among patients in the pedigree including D10S570 and D10S191, which are linked to *OPTN*. The oligonucleotide primer sequences were selected from the GDB Human Genome Database. The order and genetic distances of the markers were derived from the Marshfield Database. Microsatellites were amplified by polymerase chain reaction (PCR) following standard methods. The genotypes were obtained by silver stain and manual inspection. The pedigree and haplotype was constructed by Cyrillic version 2.1 (MathStat Software; Victoria, Australia). By searching in the NCBI database, the microsatellite marker loci, D10S570 and D10S191, near *OPTN* were selected for linkage analysis in the family. It was required that the distance between the microsatellite marker loci and *OPTN* was within a range of 2 cm and that the heterozygosity of the microsatellite marker loci was more than 0.7. D10S570 was located 0.5 cm upstream of *OPTN*, and D10S191 was located at 1.5 cm downstream of *OPTN*. The microsatellite marker was amplified by the PCR method, using the primer provided by NCBI database, and then detected by PAGE. The data processing was accomplished by the Department of Biological Information of Harbin Medical University. Reltest and Sibpal, as parts of the statistical coverall software SGEV5.2 of genetic epidemiology, were used for affected relatives analysis and slib pair linkage analysis, respectively.

### Mutation analysis

#### Extraction of DNA

From all subjects, 3 ml of blood was drawn with citric acid glucose solution anticoagulation. The white cells were disassociated and proteinase K digested. Genome DNA was extracted from peripheral blood leucocytes according to the routine method of phenol chloroform (Promega, Beijing, China) and quantitated by ultraviolet spectrophotometry.

#### Polymerase chain reaction

The primers (synthetized by Shangon Company, Shanghai, China) were designed according to the base sequence of *OPTN* exons 4−16 (See Table 1). Each 25 μl PCR reaction contained 2.5 μl 10X Buffer, 1.5 μl MgCl_2_, 2 μl dNTP, 0.5 μl TaqE (Shanghai Shangon Company), 2 μl primer(10 μmol/l), and 2 μl genomic DNA. PCR was performed on a thermal cycler with an initial denaturation step of 10 min at 95 °C followed by 35 cycles of 94 °C for 1 min, a touchdown annealing temperature of 55–60 °C for 1 min, and 72 °C for 1 min, and a final extension step of 72 °C for 5 min. All PCR products were visualized with 1.5% gel for 30 min and detected by ultraviolet spectrophotometry.

**Table 1:** *OPTN* primers used to amplify exons 4 through 16.

**Exon**	**Upstream**	**Downstream**
4	5′-GTGACTTTTCCACAGGAAC-3′	5′-GCTAAATCCTGTGCTTCC-3′
5	5′-GGTCAAGTGGACTAGAGGGAG-3′	5′-CTAGGAGTCTAGACACGTAAG-3′
6	5′-GCGATTAAGGGCATGAGCCC-3′	5′-GTTTCATCTTTCCAGGGGAGG-3′
7	5′-CTTTTGACAAAGAATTTGTCTTGT-3′	5′-GACCTCCGGTGACAAGCACCCA-3′
8	5′-AGCTGGTCCCAGTTATATTGGGT-3′	5′-GTATGGTACTTAATTATATCTCAG-3′
9	5′-ATAATTGCTATTTCTCTTAAAGCC-3′	5′-ACCCATCACAAGATTTCAATTCA-3′
10	5′-GCCTGTTTTCTCCTAAAGAGGTT-3′	5′-TATAGTAGTATACTTTGTAAAAATG-3′
11	5′-CCTTGGGGTTTGTTTAAAAGCCA-3′	5′-TGTATAAAAAGGCGATTCTCCC-3′
12	5′-TTGGGAGGCAAGACTATAAGTT-3′	5′-TTTAGTGAAGGATTCATGTAACT-3′
13	5′-CTAATATTTTACTAAAACAGGCAG-3′	5′-CACCATTGCTTTCCAATGCGAGA-3′
14	5′-GGATACAGCACTACCTCCTCATCGC-3′	5′-CGGCCATGCTGATGTGAGCTCTGGGT-3′
15	5′-TCAGTGTTGTCATGTTTCGGGGT-3′	5′-GAAGTGGAATTTTTCTTCAAGCA-3′
16	5′-GAACTGATGTTAAAACTCGCCA-3′	5′-ACCTCCCAAAGTGCTGGGATT-3′

#### Sequence analysis of PCR products

PCR products were sent to Boya Biotechnology Company (Shanghai, China)to be purified, and then two-direction sequencing was performed using an ABI 100 sequencer (Boya Biotechnology Company, Shanghai, China). The results of sequencing were compared with the GenBank original sequence using BLAST.

## Results

### Clinical evaluation

**Figure 1:**
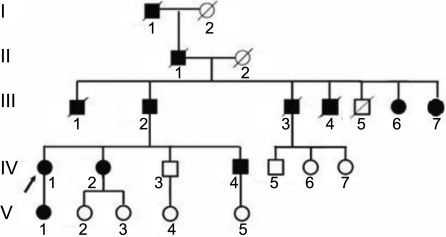
Pedigree of a six-generation family with six affected members. Blood (3 ml) was drawn from certain members (III:2, III:7, IV:1, IV:2, IV:3, IV:4, IV:5, V:1, V:2, V:3, V:4, and V:5) of the family. Solid squares mean male patients, open circles mean normal females, solid circles mean female patients, open squares mean normal males, arrow means proband, lines through circles and squares indicate deceased.

The pedigree of interest was a six-generation family with six affected members (Figure 1). Each affected member showed no gender disposition. Since the ratio of affected males to affected females was approximately 1:1, an autosomal dominant pattern of inheritance was concluded. Demographic data of 12 patients from the pedigree are shown in Table 2. After obtaining written informed consent, blood samples (3 ml) were collected from 6 affected and 6 unaffected individuals in the family pedigree. In addition, blood samples were obtained from 87 normal controls patients. Each of the blood specimens then underwent detailed investigation with the genomic DNA extracted using a standardized protocol.

**Table 2:** Demographics of 12 cases of the pedigree of interest.

**Code**	**Sex**	**Age**	**Diagnosis**	**Correct visual acuity**	**Anterior chamber**	**Angle of anterior chamber**	**Optic disc (cup/disc)**	**Intraocular pressure (mmHg)**	**Visual field**	**History of operation**
III:2	M	76	POAG	Vd:0.2; Vs:0.1	normal	open	OD:0.3~0.4; OS:0.9	Td:15.3; Ts:21.1	OU concentric contraction	Glaucoma filtered operation of OU in 1994
III:7	F	59	POAG	Vd:PL; Vs:PL	normal	open	OD:0.9; OS:0.9	Td:27.5; Ts:28.1	Can not be measured	
IV:1	F	55	POAG	Vd:0.5; Vs:0.4	normal	open	OD:0.8; OS:0.7	Td:16.0; Ts:13.0	OU concentric contraction	Glaucoma filtered operation of OU in 1995
IV:2	F	53	POAG	Vd:0.5; Vs:0.5	normal	open	OD:0.7~0.8; OS:0.6~0.7	Td:20.2; Ts:21.1	OU concentric contraction	
IV:3	M	50	POAG	Vd:0.7; Vs:0.6	normal	open	OD:0.7; OS:0.6	Td:20.1; Ts:17.5	OU concentric contraction	
IV:4	M	47	POAG	Vd:0.4; Vs:0.5	normal	open	OD:0.7; OS:0.6	Td:15.5; Ts:15.3	OU defect of nasal side	Glaucoma filtered operation of OU in 1997
IV:5	M	46		Vd:1.0; Vs:1.0	normal	open	OD:0.2~0.3; OS:0.2	Td:10.8; Ts:11.7	normal	
V:1	F	27	POAG	Vd:0.4; Vs:0.6	normal	open	OD:0.7; OS:0.7	Td:22.0; Ts:21.7	OU defect of nasal side	Glaucoma filtered operation of OU in 1998
V:2	F	26		Vd:0.6; Vs:0.5	normal	open	OD:0.5; OS:0.6	Td:18.3; Ts:17.7	normal	
V:3	F	24		Vd:1.0; Vs:1.0	normal	open	OD:0.2; OS:0.1	Td:15.7; Ts:17.3	normal	
V:4	F	21		Vd:0.7; Vs:0.8	normal	open	OD:0.3; OS:0.4	Td:18.7; Ts:18.0	OU defect of nasal side	
V:5	F	20		Vd:1.0; Vs:1.0	normal	open	OD:0.2; OS:0.3	Td:14.2; Ts:14.1	normal	

VA: Visual Acuity, refers to corrected central vision, using the international standard visual acuity chart. AC: Anterior Chamber, refers to anterior chamber depth, namely the depth from cornea to iris. C/D: cup/disc, Ratio of the diameter between optic cup and optic nerve head. IOP: Intraocular Pressure, refers to the pressure between the contents of eyeball and eyeball wall and the pressure among the contents. VF: Visual Field, refers to the scope that can be seen by the retinal photosensory cells beyond macular central fovea, when the eyes stare forward at certain point.

### Identification of PCR amplification products and analysis of DNA sequence

**Figure 2:**
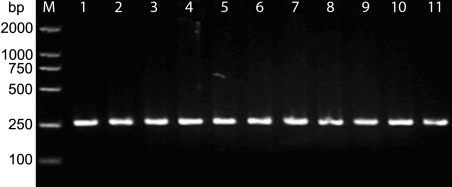
PCR products of *OPTN* exon 10. The PCR product is 260 bp in size (lanes 1-11). Lane M is the DL2000 marker.

**Figure 3:**
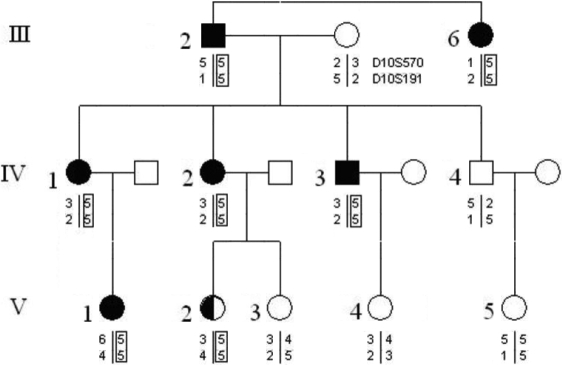
The haplotype atlas of the familial primary open angle glaucoma family. Solid squares mean male patients, open circles mean normal females, solid circles mean female patients, open squares mean normal males.

**Figure 4:**
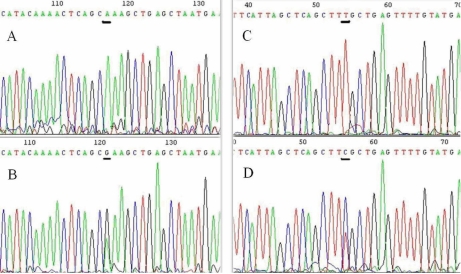
DNA sequence analysis of *OPTN* exon 10. **A**: The forward sequence of *OPTN* exon 10 in the glaucoma family. An ‘AAA’ to ‘GAA’ transition at amino acid Lys322Glu in exon 10 is observed. The underlined base is the location of mutation. **B**: The forward sequence of *OPTN* exon 10 in normal controls. **C**: The reverse sequence of *OPTN* exon 10 in the glaucoma  family. **D**: The reverse sequence of *OPTN* exon 10 in normal controls.

All the PCR amplification products were specific DNA straps, among which PCR products of exon 10 was 260 bp (See Figure 2). A LOD score ≥1 supports linkage while a LOD score ≥3 affirms the linkage, and a LOD score ≤-2 demonstrates negativing linkage. The LOD scores at D10S570 and D10S191 were 2.086101 and 1.971699, respectively, (all more than 1), indicating that the region between D10S570 and D10S191 was linked to the pathogenic gene. Allele-sharing analysis among the affected members in the family excluded linkage with the 2 loci other than those on 10p15–14 (Figure 3 and Table 3).Bi-directional sequencing was performed on all samples from affected patients and also from the controls. The sequencing results (in reverse direction) were in conformity with those in forward direction. The sequence results showed that in affected individuals III:2, III:7, IV:1, IV:2, IV:4, V:1, and V:2, an ‘AAA’ to ‘GAA’ transition at codon 322 of *OPTN* exon 10 was observed (See Figure 4). That is to say, a missense mutation A1274G in exon 10 of *OPTN* was identified in affected patients of the family. The corresponding amino acid change (codon number is 322) was lysine to glutamate (or glutamic acid). This novel mutation was not found in DNA from unaffected family members nor in DNA of the 87 unrelated normal controls.

**Table 3:** Linkage analysis of affected subjects.

**Marker**	**Affected pedigree member LOD score**	**Affected sib-pair p value**
D10S570	2.086101	0.004815
D10S191	1.971699	0.0075584

## Discussion

Epidemiology studies suggest that glaucoma is one of the leading causes of visual loss worldwide with about one hundred million patients affected and a prevalence rate of approximately 2% of the population older than 40 years of age [5]. In 1993, Sheffield and colleagues [6] undertook successful linkage analysis of DNA specimens from JOAG patients by applying small tandem repeat fragments (the correlated disease gene was localized in the region between D1S194 and D1S218 of chromosome 1). In 1997, Stone screened the candidate genes and ascertained the correct identity of the *MYOC* gene (i.e., previously known as *TIGR*).

In 1998, Sarfarazi and his team [7] performed genetic scanning on glaucoma family members with NTG, and localized the causative gene to the region between D10S1729 and D10S1664 of 10p15–14. In 2002, Rezaie and collegues [3] found that coding amino acid alterations consisted of mistranslation mutations in the *OPTN* gene.

At present, there are more than 20 mutations found in the *OPTN* coding region, four of which have close correlation with POAG. These mutations are respectively E50K of exon 4, M98K of exon 5, discontinuous protein coding of exon 6 and R545Q of exon 16 [3]. To our knowledge, this study describes the first report of a Lys322Glu mutation in exon 10 of *OPTN*. In this case, an AAA to GAA transformation at codon 1,274 in exon 10 causes an amino acid change from lysine to glutamic acid, with potential effects on protein structure and function.

*OPTN* (GenBank: AF420371, AF420372, and AF420373) is located on chromosome 10p15–14, and includes 3 non-coding exons and 13 coding exons, which are expressed in many tissues such as heart, brain, liver, skeletal muscles, kidney, especially in trabecular reticulum, non-colorant ciliary epithelium, and the retina. Optineurin, the 577 amino acid protein of *OPTN*, also called optic neuropathy inducing protein (OPTN), is a secreted protein located in the golgiosome. This protein is abundant in leucine zipper and latent zinc fingers formed by lysine-histidine-lysine. Optineurin has an effect on activating transcription factor IIIA (ATF) and Huntington, and participates the signal conveying of TNF2α. Thus, it is speculated that this protein activates apoptosis. The effectiveness of optineurin may be concerned with the conservation of optical nerves and the maintenance of intraocular tension. Consequently, it is concluded that in glaucomatous optic neuropathy, the decrease in optineurin may lead to glaucomatous defects of field vision and optic neuropathy.

The characteristics of the patients of this family: 1) autosomal dominant hereditary; 2) intraocular pressure during attack is more than 21 mmHg; 3) the patients have marked glaucoma optic disc, typical defect of visual field, and open angle of anterior chamber; 4) the age when attack occurs in the family members is inclined to be younger (the attack to III:2 was at the age of 64, the attack to IV:1 was at the age of 44, and the attack to V:1 was at the age of 22; 5) and the disease develops rapidly; 6) clinical therapeutic efficacy is relatively good: after the trabeculectomy, the range of defect of visual field decreases and intraocular pressure is within normal limits. In this family, all patients have a mutation of Lys322Glu, and at present patient V:2 has not been diagnosed with POAG. Patient V:2 also has this mutation, but the binocular visual acuity is lower than 0.6 after rectification, the nasal side disc of the left eye ground is narrowing, and the intraocular pressure and visual field are normal. Therefore a timed follow-up visit is necessary to observe the development of disease. The missense amino acid mutation could result in the alteration of protein structure and function, and influence the zinc finger structure, protein polarity and hydrophilicity, and protein transportation in cells, thus influencing protein function. This is our prediction, and we want to confirm the prediction with follow up research, which we are doing now.

In conclusion, our investigations indicate that a novel Lys322Glu mutation in *OPTN* is associated with POAG in a large family pedigree from northeastern China. Based on our analysis, an autosomal dominance inheritance pattern is present. Further studies will be needed to investigate the effect of this single base mutation on protein structure and function in this cohort of patients.
